# Winter Wheat (*Triticum aestivum* L.) Tolerance to Mulch

**DOI:** 10.3390/plants10102047

**Published:** 2021-09-29

**Authors:** Matthew R. Ryan, Sandra Wayman, Christopher J. Pelzer, Caitlin A. Peterson, Uriel D. Menalled, Terry J. Rose

**Affiliations:** 1 Section of Soil and Crop Sciences, School of Integrative Plant Science, Cornell University, Ithaca, NY 14850, USA; sw783@cornell.edu (S.W.); cjp254@cornell.edu (C.J.P.); udm3@cornell.edu (U.D.M.); 2 Water Policy Center, Public Policy Institute of California, San Francisco, CA 94111, USA; peterson@ppic.org; 3 Faculty of Science and Engineering, Southern Cross Plant Science, Southern Cross University, Lismore, NSW 2480, Australia; terry.rose@scu.edu.au

**Keywords:** cover crop, weed management, organic, no-till

## Abstract

Mulch from cover crops can effectively suppress weeds in organic corn (*Zea mays* L.) and soybean (*Glycine max* L.) as part of cover crop-based rotational no-till systems, but little is known about the feasibility of using mulch to suppress weeds in organic winter small grain crops. A field experiment was conducted in central NY, USA, to quantify winter wheat (*Triticum aestivum* L.) seedling emergence, weed and crop biomass production, and wheat grain yield across a gradient of mulch biomass. Winter wheat seedling density showed an asymptotic relationship with mulch biomass, with no effect at low rates and a gradual decrease from moderate to high rates of mulch. Selective suppression of weed biomass but not wheat biomass was observed, and wheat grain yield was not reduced at the highest level of mulch (9000 kg ha^−1^). Results indicate that organic winter wheat can be no-till planted in systems that use mulch for weed suppression. Future research should explore wheat tolerance to mulch under different conditions, and the potential of no-till planting wheat directly into rolled-crimped cover crops.

## 1. Introduction

No-till crop production has received widespread attention over the past several decades as a strategy to conserve topsoil and improve soil health while reducing fuel and labor inputs. In 2017, no-till was practiced on 26% of cropland in the United States [1]. The adoption of no-till practices since the 1980s was enabled by synthetic herbicides and improved planting equipment [2]. However, in organic production systems where synthetic herbicides are prohibited, soil tillage and cultivation are commonly used for weed management [3]. Weeds can also be suppressed by surface mulch from cover crops that are mechanically terminated with roller-crimpers, and researchers have demonstrated success with this approach for organic no-till corn (*Zea mays* L.) and soybean (*Glycine max* L.) [4]. Although cover crop-based, organic no-till production has the potential to provide some soil health benefits [5], soil tillage is typically used for establishing cover crops and small grain crops in the crop rotation. This rotational no-till approach limits the soil health benefits that manifest over a longer period (e.g., increased soil organic matter, enhanced water infiltration from preferential flow channels, etc.) [6]. Thus, research is needed to explore the potential of other crops beyond corn and soybean that can be no-till planted into mulch, which could allow for extended sequences without soil tillage in organic cropping systems.

Selective suppression of weed seedlings but not crop seedlings by mulch is important for successful cover crop-based, organic no-till production. Previous research has shown that weed suppression from mulch is a function of physical impedance and light deprivation [7] as well as chemical inhibition from allelochemicals [8]. Whereas cooler soils and reduced light transmittance from mulch tend to reduce weed emergence, mulch can also conserve soil moisture and increase weed seedling emergence, especially at low levels of mulch biomass [9]. Cover crop biomass is an important driver of weed suppression from mulch [10] and past work has suggested that 8000 kg ha^−1^ is the minimum biomass required to achieve consistent weed suppression [11]. However, this threshold likely varies by weed community as well as crop variety, environment, and management practices (i.e., G × E × M).

Weed tolerance to mulch is often correlated with seed size, where species with larger seeds are less likely to be suppressed by mulch [12]. Crop species with small seeds and light requirements for germination are generally more susceptible to suppression by mulch due to smaller nutrient reserves, among other considerations [13]. Seed size may also be the main lever that provides selective weed but not crop suppression in cover crop-based organic no-till [4,13]. Wheat seeds are smaller than corn and soybean (e.g., 0.05, 0.17 and 0.30 g seed^−1^ for wheat, soybean, and corn, respectively) [14], but still larger than many weed species [15]. As wheat is generally weed suppressive in the study region compared with corn and soybean, a lower rate of cover crop biomass might be adequate for consistent weed suppression, while avoiding suppression of wheat seedlings with lower nutrient reserves than corn and soybean. A field experiment was conducted in central New York, United States to evaluate weed suppression, wheat emergence, and wheat grain yield of two winter wheat varieties across a biomass gradient of grass-clover hay mulch. We hypothesized that weed biomass but also wheat seedling density, wheat biomass, and wheat grain yield would decrease with increasing mulch biomass. We also hypothesized that seedling emergence would vary by wheat variety and be greater in the variety marketed for its superior performance in high-residue environments.

## 2. Results and Discussion

In general, winter wheat performed well at all mulch rates, with neither wheat biomass nor grain yield suppressed by increasing amounts of mulch. Wheat seedling emergence was tolerant to mulch rates at or below 3000 kg ha^−1^ and remained at roughly 80% of the no-mulch control at mulch rates of 6000 kg ha^−1^. In contrast, weed biomass was suppressed at mulch rates above 6000 kg ha^−1^, which is lower than the mulch rates recommended for the summer annual crops (e.g., corn, soybean) where high-residue production is more commonly implemented [11]. These results suggest that minor management adjustments such as increased seeding rates could ensure acceptable winter wheat crop stands while effectively suppressing weeds in a no-till system.

### 2.1. Wheat Emergence

**Figure 1:**
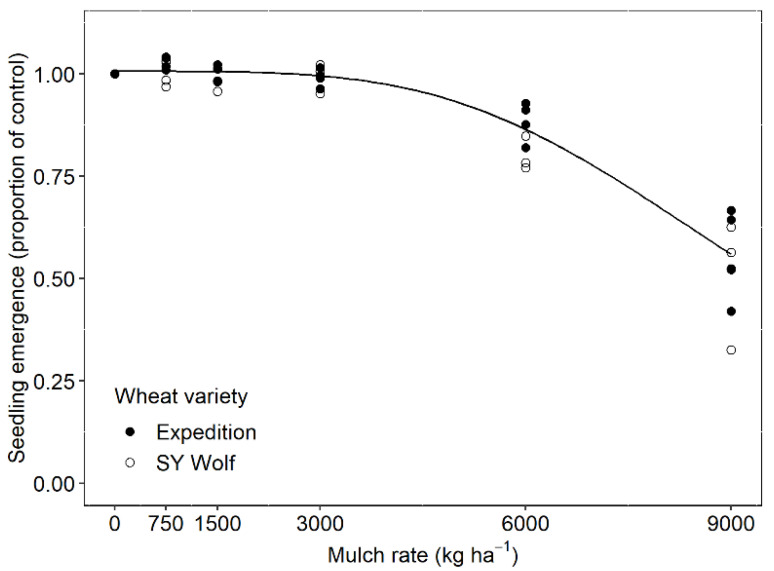
Winter wheat seedling emergence (*E*) of two winter wheat varieties, “Expedition” and “SY Wolf”, under increasing grass-clover hay mulch rates (*M*), expressed as a proportion of the emergence rate in the no-mulch control. The log-logistic response was estimated as *E* = 1.006/(1 + exp^(3.9(log(*M*) − log(9217)))^). All coefficients were significant at the α = 0.05 level, indicating a significant log-logistic response of wheat emergence to mulch rate. The response curve did not vary significantly by wheat variety (*p* = 0.26).

Wheat seedling emergence was not affected by wheat variety. When data were pooled across the two varieties, wheat seedling emergence was consistently high at low to moderate mulch rates (3000 kg ha^−1^), after which it gradually decreased to 55% of the emergence rate in the no-mulch control at the highest mulch rate (9000 kg ha^−1^; Figure 1). In addition to the lack of support for our second hypothesis about wheat varieties, the functional form of the relationship between wheat density and mulch rate was unexpected. In contrast to our results, previous experiments with broadleaf weeds spanning mulch rates like those used in our experiment have reported an exponential decline with weed seedling emergence in response to increasing mulch rate. For example, Teasdale and Mohler [7] found an exponential decline in *Amaranthus retroflexus* (redroot pigweed) emergence at increasing mulch rates.

### 2.2. Weed and Wheat Biomass

**Figure 2:**
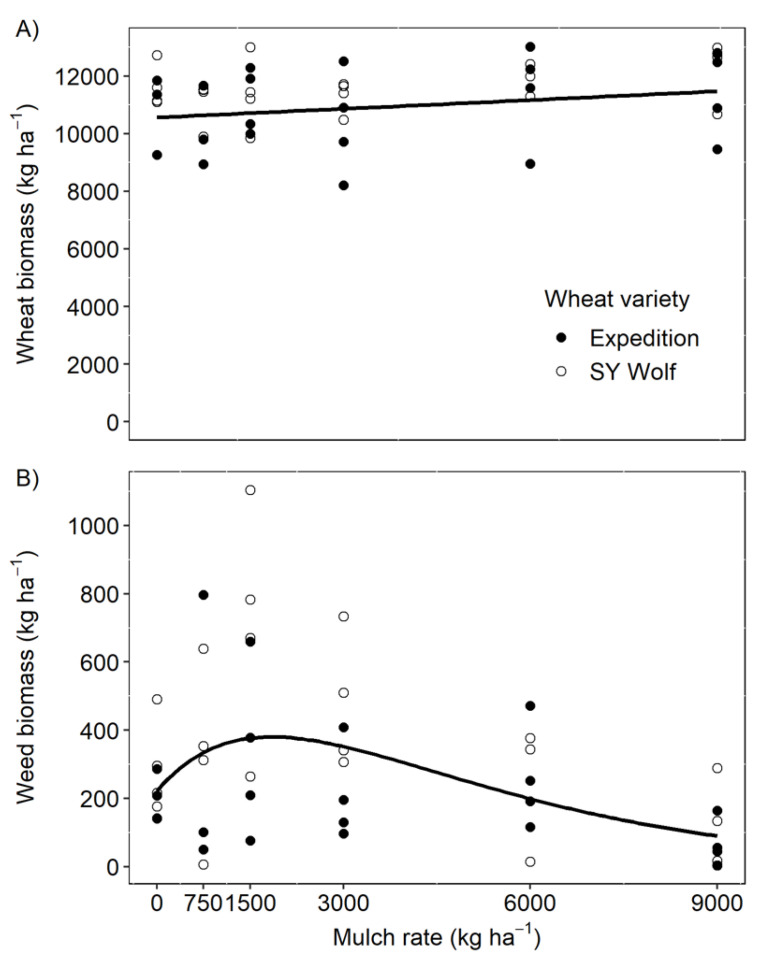
(**A**) Winter wheat biomass and (**B**) weed biomass (*W_b_*) collected from plots of two varieties of winter wheat at different grass-clover hay mulch rates (*M*). There was no significant effect of mulch rate on winter wheat biomass production (*p* = 0.06). Weed biomass response to mulch rate was estimated as *W_b_* = 222(1 + 0.0014*M*)(exp^−0.0004*M*^). All coefficients were significant at the α = 0.05 level. There was no difference in the response curves between the two varieties for either response variable (wheat biomass *p* = 0.1; weed biomass *p* = 0.1).

Wheat biomass was unaffected by mulch rate (*p* = 0.06), averaging 11,265 ± 183 kg ha^−1^ dry matter across mulch rates (stem and seed head; Figure 2A). This trend did not differ between wheat varieties (*p* = 0.18) and there was no interaction between mulch rate and wheat variety (*p* = 0.98).

In contrast to the non-effect of mulch on winter wheat biomass, we observed stimulation of weed growth at low mulch rates. Weed biomass increased from an average of 245 ± 41 with no mulch to 518 ± 122 kg ha^−1^ of biomass at a mulch rate of 1500 kg ha^−1^, but subsequently declined to a low of 89 ± 36 kg ha^−1^ biomass at the highest mulch rate of 9000 kg ha^−1^ (Figure 2B). The stimulation of weed growth at lower mulch rates was indicated by a positive mulch stimulation parameter (*a* in Equation (2); *p* < 0.001). However, at mulch rates above 2000 kg ha^−1^ weed biomass decreased (Figure 2B). Weed suppression at higher mulch biomass rates was likely due to attenuation of germination cues that reduced weed seedling density as well as light deprivation, physical interference, phytotoxin inhibition that reduced weed seedling growth [7,11]. Promotion of plant emergence at low mulch rates has long been described in the turfgrass literature, and light mulching is often used to help establish lawns [16,17]. Our results suggest that certain weed species may behave similarly to turfgrasses, with light mulch improving soil microclimate (i.e., higher and more consistent moisture, reduced temperature) in ways that promote seedling emergence and growth.

A noteworthy difference between our results and previous work on cover cropping for weed suppression in summer annual crops is that weed suppression in winter wheat was achieved at much lower biomass levels than usually recommended. A synthesis of cover cropping work in soybean and corn suggested that mulch rates of 8000 kg ha^−1^ or more are required to achieve consistent weed suppression [11]. In our study, weed biomass was not a limiting factor on wheat yield at any of the mulch rates, including rates below 8000 kg ha^−1^. Instead, wheat biomass production appeared optimal at rates of about 5000 kg ha^−1^, when the stimulatory effect of low mulch rates on weed emergence was surpassed, but wheat emergence was not yet affected. This result suggests that one of the common limitations of using cover crops for weed suppression—production of sufficient biomass—could be a smaller obstacle in a winter wheat-based system.

### 2.3. Winter Wheat Yield

**Figure 3:**
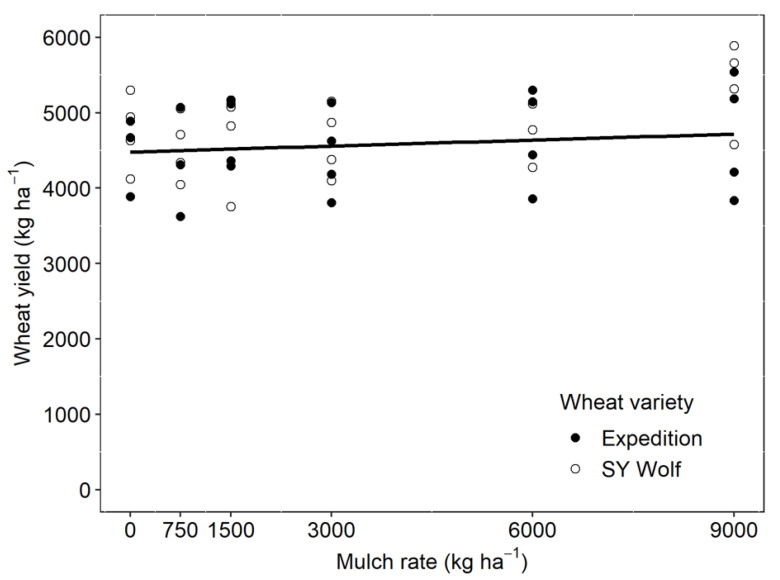
Wheat yield as affected by grass-clover hay mulch rate. There was a small positive effect of mulch rate on wheat yield (*p* < 0.05) and no difference in yield responses between wheat varieties (*p* = 0.72).

Mulch rate and wheat variety did not interact to affect wheat yield (*p* = 0.34). However, there was a slight positive trend in the response of wheat grain yield to mulch rate (*p* < 0.05; Figure 3). Across mulch rates, wheat yields averaged 4684 ± 84 kg ha^−1^, which is approximately 11% greater than average wheat yields in the region [18]. Contrary to our hypothesis, yield response to mulch rates did not differ significantly between varieties, despite the marketing of the SY Wolf variety for superior performance in high-residue conditions.

## 3. Materials and Methods

### 3.1. Site Description

**Figure 4:**
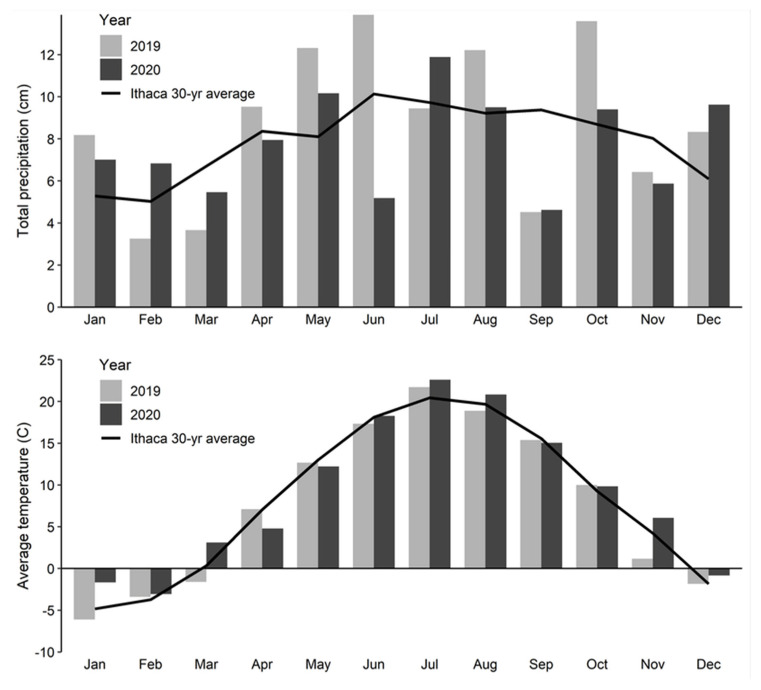
Total precipitation (cm) and average temperature (°C) by month for 2019–2020 at the Cornell Caldwell Farm (Ithaca, NY, USA). Precipitation and air temperature data were collected from an on-site weather station.

A field experiment was conducted in Ithaca, NY (42.45° N, 76.46° W) to quantify the effects of mulch mass on wheat emergence, weed suppression, and wheat yield. Soils at the site are very fine, sandy loams in the Williamson series, Typic Fragiochrept (Table 1). Prior to the experiment, the field was planted with intermediate wheatgrass (*Thinopyrum intermedium*, (Host) Barkworth & DR Dewey) in the fall of 2018 and then disked and cultipacked in fall 2019. Temperatures in 2019 and 2020 generally trended with the 30-yr normal, but precipitation in 2019 was generally higher than both the 30-yr normal and 2020 (Figure 4).

### 3.2. Experimental Design

Treatments included six mulch rates (0, 750, 1500, 3000, 6000 and 9000 kg ha^−1^ dry weight) and two hard red winter wheat varieties: (1) ‘SY Wolf’ and (2) ‘Expedition’. Expedition is commonly used by organic farmers in the northeast USA, whereas SY Wolf is a new variety marketed for good performance in heavy residue [19,20]. All wheat seed was untreated. The experimental design was a spatially balanced, split-plot randomized complete block with four blocks. Wheat variety was the main-plot factor and mulch rate was the sub-plot factor. Sub-plots measured 1 m wide × 4 m long.

### 3.3. Soil and Crop Management

The field was moldboard plowed, disked, and cultipacked several weeks prior to planting. Wheat seed was drilled to a depth of 2.5 cm into bare soil on 5 October 2019, using an Almaco heavy duty grain drill with 15 cm row spacing. Following planting, all plots were smoothed with a garden rake. Within 24 h of planting, dry grass-clover hay was weighed in the field and evenly distributed on the soil surface by hand at the six target mulch biomass rates. The grass-clover hay used as mulch was harvested from a nearby farm and consisted primarily of perennial species including orchardgrass (*Dactylis glomerata* L.), timothy (*Phleum pratense* L.), and red clover (*Trifolium pratense* L.). No amendments or fertilizer was applied to the field over the course of the experiment.

### 3.4. Data Collection

Wheat seedling emergence was documented 33 days after planting on 27 November 2019, by counting seedlings in the center four rows of each plot within a 0.5 m^2^ quadrat. On 26 July 2020, subplots were sampled by hand to determine wheat yield. In each variety by mulch rate sub-plot a 0.25 m^2^ quadrat centered over the middle 4 rows of wheat was harvested, bagged, and dried to a constant weight. Dry weights were measured for total wheat biomass as well as stem and seed head biomass. Yields are reported as threshed grain dry weights. Weeds in each quadrat were bulk harvested and dried to constant weight to determine total weed biomass, and dominant weed species in each quadrat were recorded.

### 3.5. Statistical Analysis

All data were analyzed using R version 4.0.3 [21] with packages ‘lme4′ [22], ‘nlme’ [23], and ‘stats’ [21]. We used nonlinear mixed-effects models [23] to estimate wheat seedling density and weed biomass responses to wheat variety and mulch rates. Linear mixed-effects models were used to estimate wheat biomass and wheat yield responses to mulch rate. In all models, random effects accounted for the split plot design of the experiment by nesting wheat variety within the field block. Before modeling, a Grubbs test was used to identify and remove one weed biomass and one wheat yield outlier (*p* < 0.001). Furthermore, wheat seedling density data was modeled as a proportion of seedling density in the no-mulch control (0 kg ha^−1^) within each block.

The effect of wheat variety on wheat seedling density and weed biomass was elucidated by comparing a reduced model, where data was pooled across the two wheat varieties, and a full model, where the response variables differed as a function of wheat variety. The reduced and full nonlinear models were compared using a log-likelihood ratio test. If no significant difference (*p* > 0.05) was detected between log-likelihood ratios, wheat variety had a negligible effect on the response variable. Conversely, a significant log-likelihood ratio indicated a better fit for the full model, wheat variety was considered to be a significant predictor of seedling emergence or weed biomass. If evaluation of the fitted estimates suggested violation of the assumption of homoscedacity, we fit a separate model allowing for unequal variances and compared the two fits using the same log-likelihood test. In cases where multiple nonlinear equations could reasonably be used to describe the response curve, the equation with the best fit was determined using the Akaike Information Criterion (AIC).

Wheat seedling emergence (*E*) response to mulch rate (*M*) was modeled with a 3-parameter log-logistic equation Hill equation [24]:

(1) $$E=\frac{d}{1+exp^{b\left(\log\left(M\right)-\log\left(e\right)\right)}}$$

where *d* is the upper asymptote of seeding emergence, *b* is the slope at the inflection point, and *e* is the mulch rate halfway between the upper and lower asymptote of seedling emergence.

The change in weed biomass (*W_b_*) across the mulch biomass (*M*) gradient was modeled with a right-skewed, hump-shaped equation devised by Teasdale and Mohler (2000):

(2) $$W_{b}=W_{0}\left(1+aM\right)(exp^{-bM})$$

where *W*_0_ represents the intercept, *a* represents the mulch stimulation effect, and *b* represents the mulch suppression effect.

For the linear mixed-effects models, mulch rate, wheat variety, and the interaction of these two predictor variables were fixed effects. Weed biomass did not affect wheat yield or biomass (*p* ≥ 0.50) and was not included as a predictor variable in the linear mixed models. Denominator degrees of freedom were calculated using the Kenward-Roger method and the linear mixed effects models were assessed through type III ANOVA tests.

## 4. Conclusions

We compared the effects of increasing mulch biomass on weed suppression and winter wheat performance and our results suggest wheat is relatively tolerant to mulch. The wheat seedlings in our experiment were subject to more extreme emergence restrictions than usual because mulch was placed on top of wheat seeds after seeding into bare ground, thus reducing any aid to emergence created by coulters slicing through the mulch during planting. Although wheat seedling density was reduced at high levels of mulch, wheat biomass and grain yield were tolerant to increasing mulch. On the other hand, weed biomass was stimulated at low levels and suppressed at high levels of mulch.

Given the differential tolerance to mulch, wheat may be a viable candidate for no-till planting into rolled-crimped cover crops. More research is needed across different environments, wheat varieties, and types of mulch to understand the full potential of organic no-till wheat production and how multiple organic no-till cash crops can be combined in a rotation. Such extended sequences of no-till production could provide enhanced soil health benefits.

## Figures and Tables

**Table 1:** Selected soil properties from the field site at the Caldwell Farm at Cornell University in Ithaca, NY, USA. Soil samples (20 cm depth) were analyzed by Dairy One Agronomy Services, Ithaca, NY, USA. Cations were measured using the Morgan method.

Soil Property	Value
pH	6.1
Organic matter (%)	2.6
Phosphorus (kg ha^−1^)	6.7
Potassium (kg ha^−1^)	141
Calcium (kg ha^−1^)	2180
Magnesium (kg ha^−1^)	278
Iron (kg ha^−1^)	26
Manganese (kg ha^−1^)	23
Zing (kg ha^−1^)	0.45
Aluminium (kg ha^−1^)	88

## Data Availability

The datasets generated during and/or analyzed during the current study are available from the corresponding author on reasonable request.
